# A survey on the state of the art of force myography technique (FMG): analysis and assessment

**DOI:** 10.1007/s11517-024-03019-w

**Published:** 2024-02-02

**Authors:** Omar Sherif, Mohamed Mahgoub Bassuoni, Omar Mehrez

**Affiliations:** Mechanical Power Engineering Department, Faculty of Engineering, Tanta University, Tanta, Egypt

**Keywords:** Biofeedback, FMG, EMG, Machine learning, Robotic exoskeletons, Feature Extraction

## Abstract

Precise feedback assures precise control commands especially for assistive or rehabilitation devices. Biofeedback systems integrated with assistive or rehabilitative robotic exoskeletons tend to increase its performance and effectiveness. Therefore, there has been plenty of research in the field of biofeedback covering different aspects such as signal acquisition, conditioning, feature extraction and integration with the control system. Among several types of biofeedback systems, Force myography (FMG) technique is a promising one in terms of affordability, high classification accuracies, ease to use, and low computational cost. Compared to traditional biofeedback systems such as electromyography (EMG) which offers some invasive techniques, FMG offers a completely non-invasive solution with much less effort for preprocessing with high accuracies. This work covers the whole aspects of FMG technique in terms of signal acquisition, feature extraction, signal processing, developing the machine learning model, evaluating tools for the performance of the model. Stating the difference between real-time and offline assessment, also highlighting the main uncovered points for further study, and thus enhancing the development of this technique.

**Graphical abstract:**

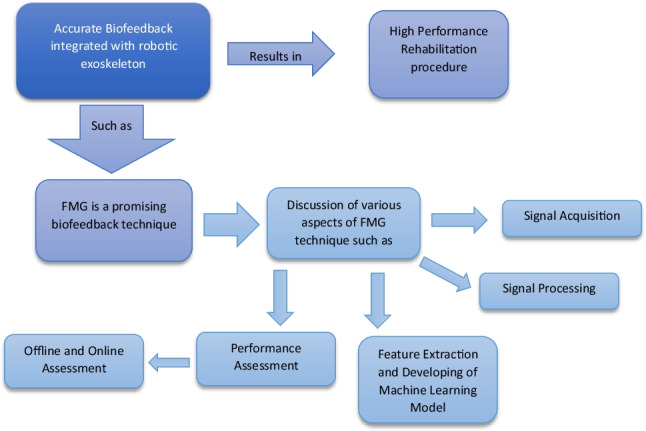

## Introduction

Motor impairments associated with neurological disorders such as stroke, spinal cord injury, and Cerebal palsy can lead to disability or neuromotor dysfunction [102]. As a result, those people will not be able to perform any activities of daily living (ADLs) as their limb neurologically cannot work well. Repetitive movement rehabilitation is delivered to improve function leveraging on brain and spinal cord plasticity [78].

Many therapists’ centers adopt some traditional rehabilitation schemes. Unfortunately, these schemes may not be appropriate for all cases. In addition, the number of such cases is growing, and the duration of treatment is long.

Exoskeleton robots contribute to fulfilling the rehabilitation goals [81]. Robotic Exoskeletons equipped with biofeedback systems can perform some repetitive exercises which are considered an effective kind of rehabilitation especially, for patients with post-stroke impairments [52, 101]. Biofeedback refers to any method in which an external device provides information on an individual's physiological responses [61]. Biofeedback measurement systems can detect human intention and thus provide an assistive action for the patient, which in certain conditions plays a vital role in the rehabilitation process. An exoskeleton used for an assistive application, when the patient intends to move his hand to grasp something, the exoskeleton detect his intention and provides an appropriate assistive action, as his hand has no neural problems [95]. In such cases, wrong or even slow responses of such exoskeletons can cause serious problems, especially for real-time applications.

**Fig. 1:**
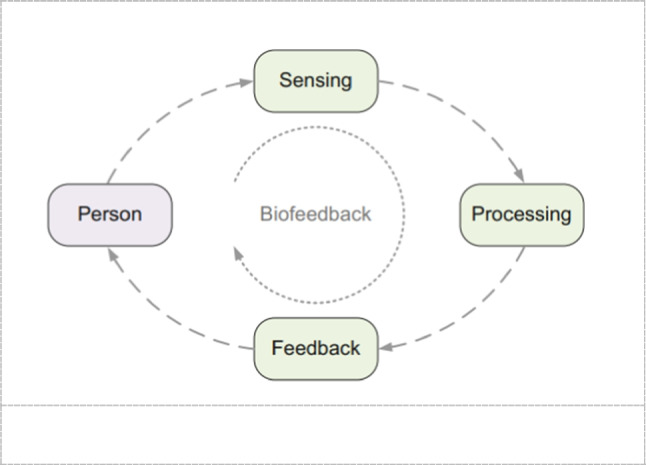
Cyclic process of a biofeedback system [55]

Biofeedback systems can also be used as diagnostic systems to capture some related data e.g., for the muscular activity. It provides useful information for therapists to decide which rehabilitation scheme the patient has to do. This is critical information since some patients are not with the same damage level. Biofeedback signals can give an indication about how efficient the rehabilitation process was. A cyclic process with elements or functional blocks that are performing sensing, processing, and giving feedback is shown in Fig. 1.

It is clear the importance of biofeedback systems that are integrated into such exoskeletons [95]. Previous research demonstrated the advantages of biofeedback improving abnormal muscle activation and promoting motor recovery [8]. Consequently, it has been integrated in robotic exoskeletons used for rehabilitation [44, 56, 99, 104, 117].

**Fig. 2:**
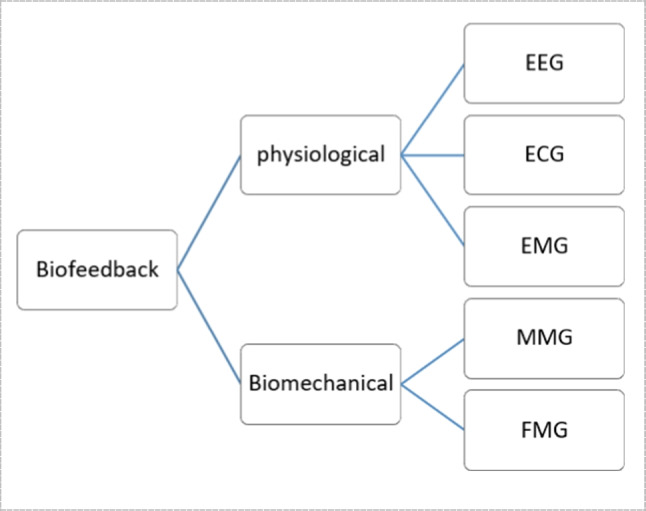
Types of biofeedback systems

Biofeedback systems can be categorized, as shown in Fig. 2, into two main categories, physiological and biomechanical biofeedback [38]. The classification is based on the type of data being measured and the goals of the biofeedback system. Physiological biofeedback deals with internal physiological processes, while biomechanical biofeedback focuses on external mechanical aspects of body movement and posture.

Physiological feedback is connected to physiological processes. It is based on signals and parameters acquired from neuromuscular system, cardiovascular system, respiratory system, brain, skin, and other body systems. Examples are electrocardiogram (ECG), electroencephalography (EEG) [9, 10, 64], electromyography (EMG) and others. For biomechanical feedback, it is classified based on signals and characteristics derived from measurements of body or body component motions. For instance, Mechanomyography (MMG) is used to detect muscle contractions by mechanical vibrations, whereas the more recent technology Force Myography (FMG) examines muscle activity by measuring the forces or pressures created by muscle movements. Signals measured by these approaches are categorized as follows: MMG collects mechanical vibrations, whereas FMG records force data, making both technologies useful for different biomechanical examinations.

Not all biofeedback systems are suitable for all conditions since every biofeedback system has its own pros and cons. For cost-dependent or technical reasons some biofeedback systems are preferred above others [8]. FMG is a trendy biofeedback technique due to its low-cost and feasibility. Also, it requires low-level of signal processing since FMG signals are not affected by interference by other electronic components. It requires low sampling rate compared to many other techniques as MMG, EMG and EEG. As a result, it has been adopted in many literatures in either predicting hand gestures or the exerted torque or even studying the effect of certain configurations, giving high accuracies in most cases [21, 46, 87, 90, 118].

There were not enough reviews covering the main aspects of FMG technology except for [111]. In this paper, the whole aspects of FMG technique will be highlighted, covering the whole procedure starting from signal acquisition to generating the predictive machine learning model.

In this paper, the concept of FMG as a biofeedback system will be introduced. In Sect. 2, it will be identified how to acquire signal data from a FMG sensor, its types and placement. Section 3 is concerning with the signal processing for FMG. Performance evaluation parameters will be discussed in Sect. 4. Discussion and challenges are introduced in Sect. 5. Finally, conclusions are given in Sect. 6.

### FMG technology

**Fig. 3:**
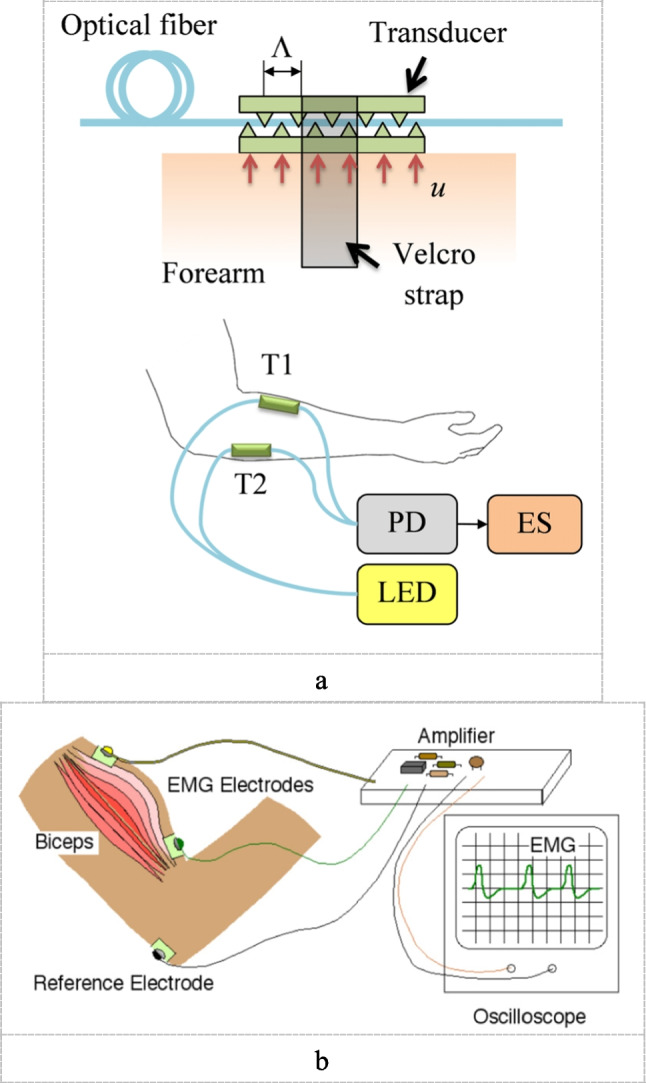
**a** Force myography principal [30]. **b** Electromyography principle [67]

Force myography (FMG) or residual kinetics is a unique way of detecting functional or neurological motor activity by measuring volumetric changes in muscles [68, 91]. It is a non-invasive technique to decode the position or movement of the muscle and convert it into a measurable signal Fig. 3a. In addition, force myography can be used to present a pressure mapping for a certain group of muscles for better understanding the effect of certain configurations.

One type of signal acquisition tools of FMG is force sensitive resistors. Its produces signals related to the volumetric changes. The more the volumetric change is, the more the resistance of the semi-conductor material goes down and hence a signal is generated which is related to the physical process the patient is undergoing. Later this signal is used for further processing [34]. By tracking the volumetric patterns associated with the contraction of a group of muscles for a group of patients, a predictive machine learning model can be developed to either predict the patient intention or to estimate the required torque for a certain situation [21].

One type of biofeedback techniques is Electromyography (EMG) Fig. 3b. EMG signals are generated by the electrical activity of the muscle fibers active during a muscle contraction [69]. These signals have been used to track muscle activity, motor function, and figure out muscle fatigue [1, 20, 70, 103]. It can also be used for developing predictive machine learning models. Although, it achieves high classification accuracies in predicting gestures, but it requires a high computational cost, skin preparation, and high-cost tools. On the other side, FMG has the advantages that made it as an alternative technique in recent years.

FMG is considered as the mechanical counterpart of EMG, it does not require precise sensor placement or extensive skin preparation [111–116]. On the contrary, EMG electrodes require to be placed at certain positions on the targeted group of muscles, also the skin must be prepared with some alcohol to remove any sweat or pollutants that may affect the signal acquisition [71, 103].

A critical point is that FMG does not require the same level of signal processing as for EMG signals which may be affected by the interference of electronic components [64]. FMG signals requires less complex instrumentation and a lower sampling rate than the higher bandwidth EMG signals [100]. Furthermore, FMG is a more affordable alternative to other muscle activity tracking methods [14, 108]. For the abovementioned reasons, it is clear why FMG gained a lot of interest from the research community [116], especially in the last ten years as a promising, affordable, easy-to-use technology**.**

## Signal acquisition

Wrong signal acquisition means wrong input data for the controller and hence wrong control commands. So, extremely high attention must be given to how these signals are collected and processed. Although FMG has the advantage over many biofeedback techniques, there are certain choices which are dependent on the application and precise requirements [108, 111].

Volumetric changes of a muscle need to be converted into a measurable signal. This led to a variety of sensors with different methods for transforming these volumetric changes into digital signals. The converting process is dependent on the type of sensor and its principle of operation.

In this section, several types of sensors used to measure FMG signals will be discussed, then methods for signal conditioning in addition to the effect of the number of sensors and their placements will be presented. Finally, some related applications using FMG technique will be mentioned.

### Sensor types

Most FMG sensors are based on resistive polymer-thick film (RPTF) technology [111], which contains force sensing resistors (FSR), either configured in a matrix [114] or array forms [22]. Other types include capacitive-optical fiber, textile, piezoelectric, and pneumatic based sensors. Some of these types are discussed below.

1. For RPTF, a FSR have two terminals of variable resistance elements that respond to force applied to the sensing area. Generally, they are comprised of a conductive polymer material pressed between two electrode layers, giving it the ability to electrically respond to volumetric changes of the muscle. Resistance of the sensor is inversely proportional to the applied force. The more the applied pressure, the lower the resistance and hence an electric signal related to the physical phenomena is generated. When there is no pressure acting on the sensor, it acts like an open circuit.

FSRs configurations can be categorized based on the response characteristics as one of two types: Shunt mode and Thru mode. These two configurations have the same function, but they exhibit different behaviors on the response curve. The Thru mode gives a steeper and less linear curve [92].

- Shunt mode configuration

**Fig. 4:**
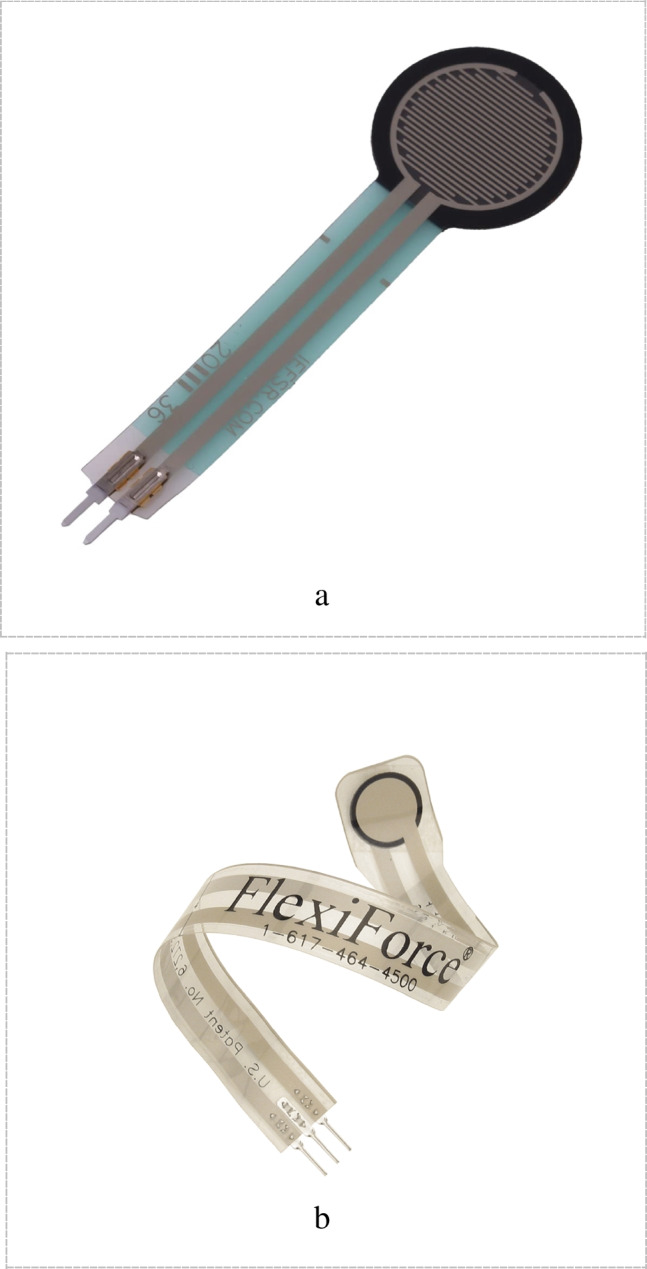
**a** Construction of a Shunt mode sensor. **b**. Flexiforce sensor

In this configuration, the top layer of the FSR is a solid region of a semiconductive FSR element placed on a flexible substrate. The bottom layer is made up of electrical traces organized in two sets of interdigitating fingers on a flexible substrate [92] Fig. 4a.

b. Thru mode configuration

In a Thru mode configuration, a solid semiconductive FSR element is placed on top of a solid conductive region, entirely covering the conductor. This is repeated on both the top and bottom layers, which are then bonded together. The semiconductor layer is sandwiched between two flat electrodes, Fig. 4b [98].

**Fig. 5:**
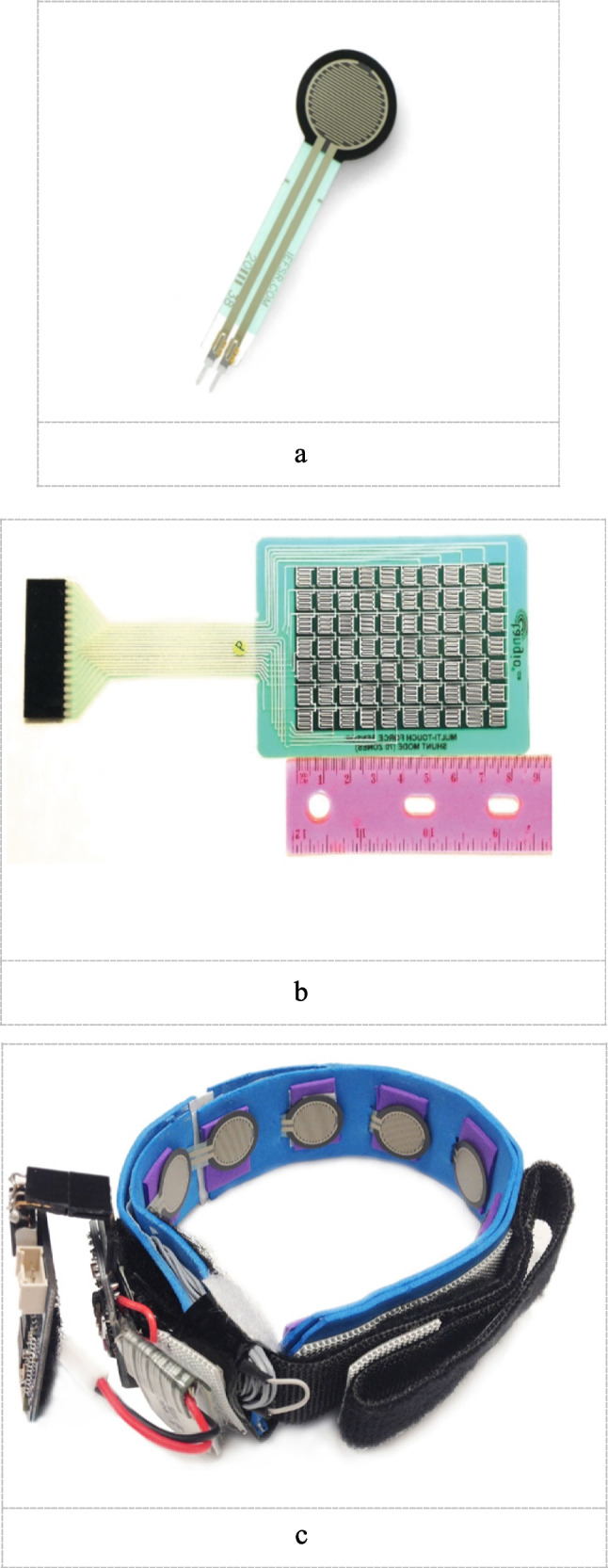
**a** Single-Zone FSR. **b** FMG matrix [114]. **c** FSR strap [22]

Also, FSRs Configuration may be categorized into: Single-Zone FSR, Array of FSRs, Matrix form as shown in Fig. 5a, b and c. The basis for this classification is the physical arrangement and connectivity of the force-sensitive resistors. For High-Density Polymer Thick Film Sensor Matrix, such a configuration is used to provide a more accurate FMG signals for the volumetric change of the muscle or make pressure mapping for a certain group of muscles. As discussed by Carlo et al. [114], Tangio Printed Electronics' TPE-901 multi-touch sensor pad was utilized as the main sensing element to record the High-density Force Myography (HD-FMG) signals. It includes a total of 70 sensing components that span an active sensing area of around 8 cm by 5.2 cm. Each sensing element is 0.5 cm by 0.5 cm in size, with a 0.2 cm gap between neighboring sensors. This HD-FMG matrix used to make a pressure mapping in the distal end of the forearm to discuss the effect of the forearm rotation on FMG signals extracted from the wrist. It is worth mentioning that the HD-FMG matrix allows for exact monitoring of muscle stiffness changes, improving sensitivity in recording FMG signals, which is critical for recognizing hand motions across different forearm postures.

2. Capacitive-optical fiber-based sensors

**Fig. 6:**
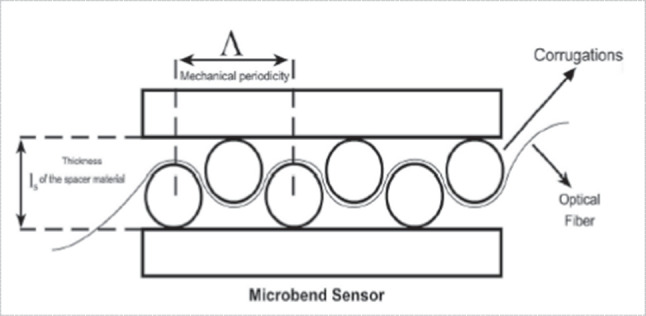
Optical fiber sensor

Optical fiber sensors (OFS) are considered as an alternative option for biomechanical measures due to inherent qualities such as flexibility, lightweight, high sensitivity, distributed sensing capacity, and immunity to electromagnetic interference [36]. As shown in Fig. 6, the sensing region is made up of two corrugated plates. Corrugations are cylindrical rods with predetermined sizes. By applying varying pressures to the top plate, the fiber is pushed between these plates. These corrugated plates carry optical fiber [28].

3. Piezoelectric based sensors

**Fig. 7:**
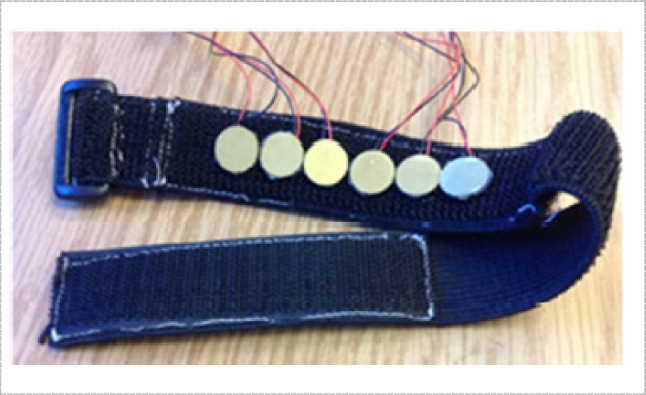
Piezoelectric sensor

Piezoelectric sensors do not generate an electric signal due to muscle deformation, but they measure it indirectly, by generating a voltage proportional to changes in applied pressure on the sensor [13]. The pressure differential induced by tendon deformation can be utilized to detect finger movements when placed tightly against the skin using a wrist strap as shown in Fig. 7. This type of sensors is highly sensitive and have minimal noise [15]. Also, Piezoelectric sensors voltage is linearly proportional to the applied pressure.

### Signal conditioning

**Fig. 8:**
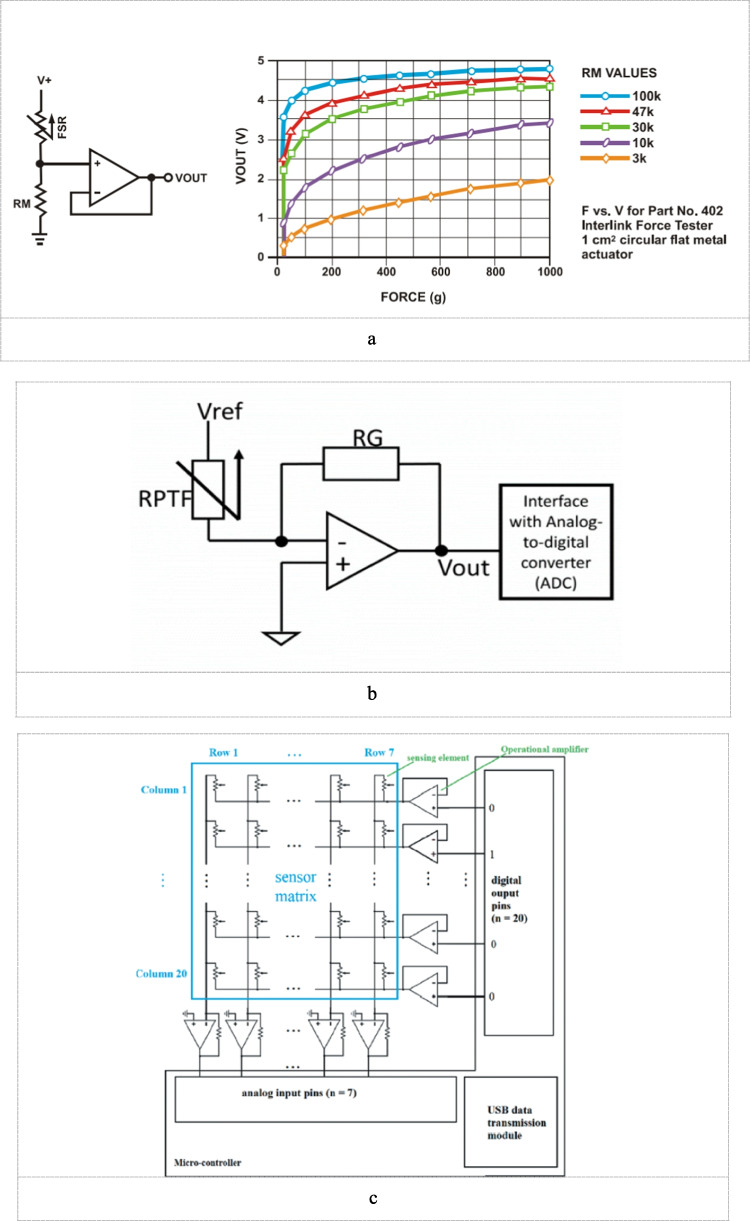
**a** FSR voltage divider circuit [12]. **b** Current to voltage setup for FSR voltage divider circuit [111]. **c** Line Scanning circuit for HD-FMG matrix using operational amplifiers [114]

A voltage divider is the most commonly used circuitry to extract the signal from the FSR sensor. It offers a better solution in terms of simplicity and ease of setup. It requires only one bias resistor (RB) to condition the output voltage of the FSR sensor [21, 88]. The output of the voltage divider is then amplified by an op-amp with a unity gain configuration and then is converted to a digital value by an ADC Fig. 8a.

The main drawback of this circuitry is the non-linear characteristics of the output voltage which complicates the sensor drift characteristic [29]. A current-to-voltage setup can be used to reduce the nonlinearity of response by using an inverting op-amp and a resistor for controlling the output gain Fig. 8b, [72, 75].

For a Matrix configuration of FSR, another circuitry called line scanning method is used to turn the sensors on and off sequentially and only allows the signal from one sensor at a time to be fed to the input terminal. Every sensor has two terminals, one connected to the common corresponding row line and the other to the common column line. Only one of the digital pins is set to “HIGH” and hence the sensor readings of the corresponding column would be read through analog input pins.

A problem that commonly occurs in such circuitry is the crosstalk phenomenon. It means for a certain matrix, when more than one sensing element is pressed, each element affects the other resulting in a lower reading for the actual force. Such a phenomenon occurs due to current leaked within the sensor network and it can be combated by using inverting operational amplifiers (op-amp) with unity feedback as shown in Fig. 8c. This will condition and stabilize the signal reading as discussed in [107].

### Sample rate

The sampling rate is the number of data points recorded every second. Many works of literature used 10 Hz as a sampling rate [21, 88, 90, 117]. It is also dependent on the application, according to [111], it ranges from 6 to 1000 Hz. For Example, static hand gestures require lower sampling rates compared to dynamic actions. A lower sampling rate, with the case of a dynamic action, will lead to loss of information and hence introduce errors. Also, whether the type of action was isometric (static) or dynamic, the root mean square error (RMSE) decreases as the sample rate increases, [112]. Concluding that, the minimal sampling frequency capable of capturing the signal features is the appropriate sampling frequency.

### Number of FSR

Number of sensors is an important issue that need to be considered in designing the sensing element, which may be an array or a matrix of sensors. Defining the appropriate number depends on a lot of factors. Some works of literature used an array or matrix of FSRs to provide a precise capture of muscle activity. The work presented by Castellini et al., [18] utilized the high-density force-sensing matrix approach to predict wrist and finger movements, while other uses only a strap with around 8–16 FSR at defined locations [21, 88]. Got`

As claimed by [111], a voltage divider can be used as an extracting circuitry for eight FSR or less in a strap, while as for more than eight FSR sensors the line scanning method is suggested. However, the work presented by Rana Chengani et., [21] uses sixteen FSR in a strap with a voltage divider as an extracting circuitry for detecting different hand gestures obtaining high accuracies in detection.

In summary, there is no direct rule for identifying the number of sensors required for a certain application. The number of sensors depends on precise requirements, the muscle of interest that its activation need to be tracked, and the affordability. Placement of the sensor is an important factor that needs to be considered as will be discussed in the next section.

### Sensor placement and applications

As mentioned above a matrix of FSR can provide more precise data, but if a strap is placed in an optimal location for a certain gesture to be detected then we will have accuracies similar to a matrix of FSRs located at the same location as discussed by [21]. Depending on the required gestures or movements to be detected or the studied muscle, identifying the optimal location and number of sensors could provide a low-cost solution with high accuracy results.

While our primary emphasis is on FMG, it's crucial to acknowledge that the importance of sensor location is a shared consideration among different muscle activity monitoring techniques. For example, in a different technique such as MMG, Cescon et al. [19] studied the impact of sensor location along fibers on MMG signal characteristics in various muscles using 8 unidirectional accelerometers. The study revealed that MMG amplitude and frequency content are highly dependent on the location of the detection device. This conclusion demonstrates the crucial role of a sensor location on results obtained.

Most of literature are targeting the detection of upper limb movements from the musculotendinous complex of the forearm. In applications for users with intact limbs, researchers use an array of FMG sensors either at the belly muscle at the forearm, or near the wrist, or both. For summary, in Table 1, numerous works are mentioned with their applications.

**Table 1:** Research works with different applications

Reference	Name of publication	Application
[7]	Wrist Force Myography (FMG) Exploitation for Finger Signs Distinguishing	Hand gesture Detection
[3]	Hand gesture recognition using force myography of the forearm activities and optimized features	Hand gesture Detection
[6]	Four Sensors Bracelet for American Sign Language Recognition based on Wrist Force Myography	Hand gesture Detection
[22]	Force Myography to Control Robotic Upper Extremity Prostheses: A Feasibility Study	Hand gesture Detection
[121]	Deep Learning Technique in Recognizing Hand Grasps using FMG signals	Hand gesture Detection
[63]	Combined Use of FSR Sensor Array and SVM Classifier for Finger Motion Recognition Based on Pressure Distribution Map	Hand gesture Detection
[117]	Design of a wearable FMG sensing system for user intent detection during hand rehabilitation with a soft robotic glove	Hand gesture Detection
[42]	Performance of Forearm FMG for Estimating Hand Gestures and Prosthetic Hand Control	Hand gesture Detection
[75]	Using FSR based muscle activity monitoring to recognize manipulative arm gestures	Hand gesture Detection
[2]	Toward Intuitive Prosthetic Control: Solving Common Issues Using Force Myography, Surface Electromyography, and Pattern Recognition in a Pilot Case Study	Hand gesture Detection
[53]	Continuous Prediction of Finger Movements Using Force Myography	Hand gesture Detection
[96]	Wearable Tactile Sensor Brace for Motion Intent Recognition in Upper-Limb Rehabilitation	Hand gesture Detection
[34]	Hand Gesture Recognition Based on Force Myography Measurements using KNN Classifier	Hand gesture Detection
[46]	Exploration of Force Myography and surface Electromyography in hand gesture classification	Hand gesture Detection
[51]	A novel, co-located EMG-FMG-sensing wearable armband for hand gesture recognition	Hand gesture Detection
[23]	Machine-learning-based hand motion recognition system by measuring forearm deformation with a distance sensor array	Hand gesture Detection
[84]	A wearable sensor system for rehabilitation applications	Hand gesture Detection
[11]	FMG vs EMG: A Comparison of Usability for Real-time Pattern Recognition Based Control	Hand gesture Detection
[13]	A Wrist-Worn Piezoelectric Sensor Array for Gesture Input	Hand gesture Detection
[50]	Virtual grasps recognition using fusion of Leap Motion and force myography	Hand gesture Detection
[58]	Shape conformable high spatial resolution tactile bracelet for detecting hand and wrist activity	Hand gesture Detection
[26]	Investigation into the Potential to Create a Force Myography-based Smart-home Controller for Aging Populations	Hand gesture Detection
[5]	A Machine Learning Processing Pipeline for Reliable Hand Gesture Classification of FMG Signals with Stochastic Variance	Hand gesture Detection
[72]	A Smart Watch with Embedded Sensors to Recognize Objects, Grasps and Forearm Gestures	Hand gesture Detection
[35]	Optical Fiber Force Myography Sensor for Identification of Hand Postures	Hand gesture Detection
[33]	A Case Study of a Force-myography Controlled Bionic Hand Mitigating Limb Position Effect	Hand gesture Detection
[31]	Fabrication, Structure Characterization, and Performance Testing of Piezoelectric-Film Sensors for Recording Body Motion	Hand gesture Detection
[16]	Using a high spatial resolution tactile sensor for intention detection	Hand gesture Detection
[77]	Real-time classification of simultaneous hand and wrist motions using Artificial Neural Networks with variable threshold outputs	Hand gesture Detection
[86]	Regressing grasping using force myography: An exploratory study	Hand gesture Detection
[90]	A compact robotic orthosis for wrist assistance	Wrist Rehabilitation
[113]	Does force myography recorded at the wrist correlate to resistance load levels during bicep curls?	Wrist Rehabilitation
[87]	On the estimation of isometric wrist/forearm torque about three axes using Force Myography	Wrist Rehabilitation
[25]	Wrist-worn wearables based on force myography: on the significance of user anthropometry	Wrist Rehabilitation
[114]	Towards the investigation on the effect of the forearm rotation on the wrist FMG signal pattern using a high-density FMG sensing matrix	Wrist Rehabilitation
[54]	Improving the Robustness of Human–Machine Interactive Control for Myoelectric Prosthetic Hand During Arm Position Changing	Prosthetic Control
[74]	Multi-modal myocontrol: Testing combined force- and electromyography	Prosthetic Control
[14]	An Innovative Multisensor Controlled Prosthetic Hand	Prosthetic Control
[41]	Force Myography Signal-Based Hand Gesture Classification for the Implementation of Real-Time Control System to a Prosthetic Hand	Prosthetic Control
[80]	High-density force myography: A possible alternative for upper-limb prosthetic control	Prosthetic Control
[105]	Biomechatronic approach to a multi-fingered hand prosthesis	Prosthetic Control
[82]	Stable force-myographic control of a prosthetic hand using incremental learning	Prosthetic Control
[37]	A Multi-sensor Approach for Biomimetic Control of a Robotic Prosthetic Hand	Prosthetic Control
[4]	FMG- and RNN-Based Estimation of Motor Intention of Upper-Limb Motion in Human–Robot Collaboration	Upper Limb
[18]	Tactile Myography: An Off-Line Assessment of Able-Bodied Subjects and One Upper-Limb Amputee	Upper Limb
[85]	Force Myography for Monitoring Grasping in Individuals with Stroke with Mild to Moderate Upper-Extremity Impairments: A Preliminary Investigation in a Controlled Environment	Upper Limb
[123]	A Pilot Study on Using Force myography to Record Upper-limb Movements for Human–machine Interactive Control	Upper Limb
[108]	Towards the development of a wearable feedback system for monitoring the activities of the upper-extremities	Upper Limb
[110]	Performance of Forearm FMG and sEMG for Estimating Elbow, Forearm and Wrist Positions	Upper Limb
[94]	Assessment of Low-Density Force Myography Armband for Classification of Upper Limb Gestures	Upper Limb
[65]	FMG-Based Body Motion Registration Using Piezoelectret Sensors	Lower limb
[40]	Force Myography Based Novel Strategy for Locomotion Classification	Lower limb
[39]	Locomotion mode classification using force myography	Lower limb
[59]	Extreme Learning Machine Classification Method for Lower Limb Movement recognition	Lower limb
[47]	Ankle positions classification using force myography: An exploratory investigation	Ankle rehabilitation
[43]	Building Effective Machine Learning Models for Ankle Joint Power Estimation During Walking Using FMG Sensors	Ankle rehabilitation
[28]	Prediction of force measurements of a microbend sensor based on an artificial neural network	Force prediction
[119]	Estimating Exerted Hand Force via Force Myography to Interact with a Biaxial Stage in Real-Time by Learning Human Intentions: A Preliminary Investigation	Force prediction
[17]	A wearable low-cost device based upon Force-Sensing Resistors to detect single-finger forces	Force prediction
[120]	Toward Long-Term FMG Model-Based Estimation of Applied Hand Force in Dynamic Motion During Human–Robot Interactions	Force prediction
[88]	Regressing force-myographic signals collected by an armband to estimate torque exerted by the wrist: A preliminary investigation	Force prediction
[106]	Pressure signature of forearm as predictor of Grip Force	Force prediction
[115]	Optimization of Force myography Sensor Placement for Arm Movement Recognition	Sensor placement
[21]	Pilot study on strategies in sensor placement for robust hand/wrist gesture classification based on movement related changes in forearm volume	Sensor placement
[49]	A wearable gait phase detection system based on force myography techniques	Gait Detection
[48]	Exploration of gait parameters affecting the accuracy of force myography-based gait phase detection	Gait Detection

**Fig. 9:**
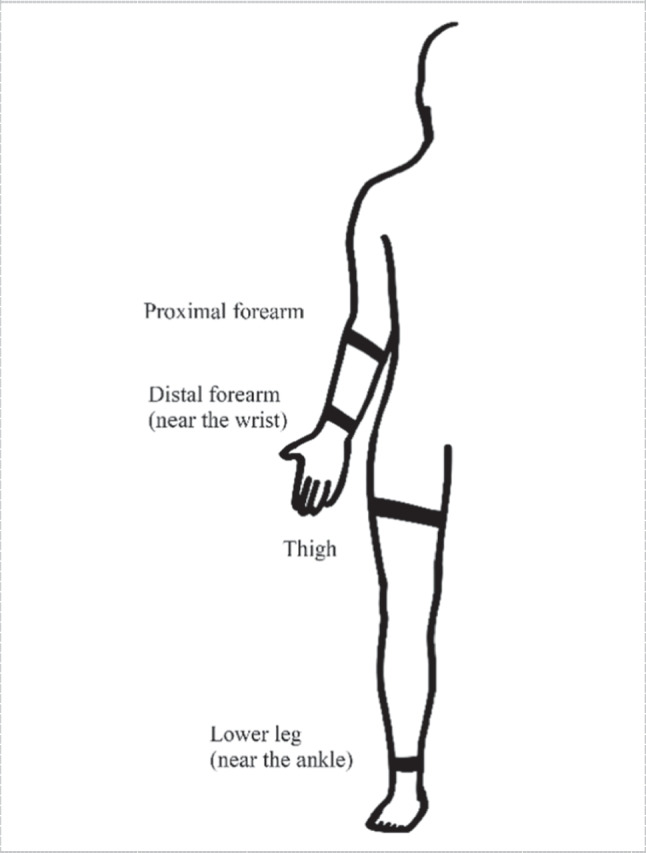
Common locations for FSR straps in bulk of forearm, distal forearm, wrist, and some may be located at the upper limb [111]. Also, for thigh and ankle are common positions

Signals extracted from these regions are used to predict hand gestures or finger movements [13, 50, 84, 109, 115, 117]. As presented by Jiang et al. [46] showed that 48 hand gestures could be predicted from one of the two previously mentioned positions, achieving an average of 90% cross-trial validations accuracy. These 48 gestures were 16 grasp types, 16 sign language gestures, and 16 finger and hand movements. Also, Yunger et al. used FSR sensors in a sleeve that covered the whole forearm to predict grip force in a stroke rehabilitation experiment to improve fine motor function [118]. Some of common locations for FSR straps as in Fig. 9.

**Fig. 10:**
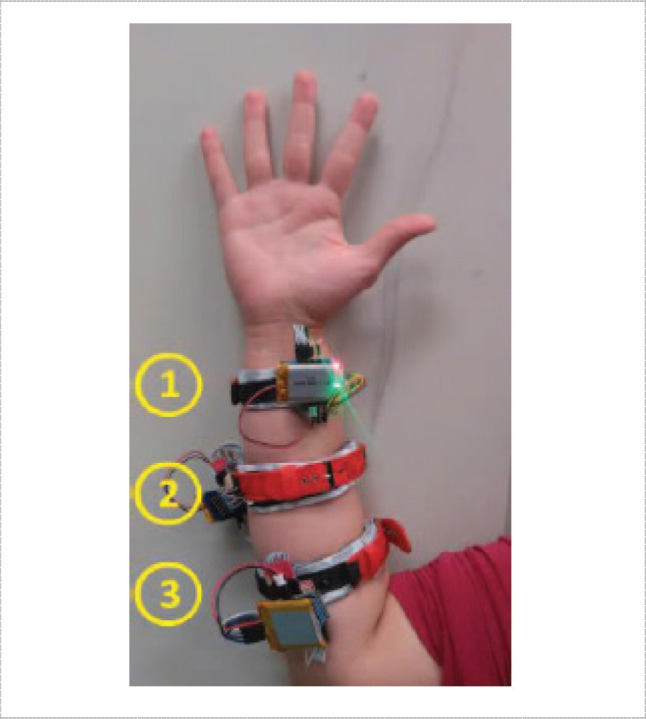
Placement of FMG array strap in three positions: (1) 2.25 cm proximal to the wrist, (2) midway between position 1 and 3, (3) at the bully muscle which has the widest circumference [21]

In [90] Sangha et al., presented a compact wrist exoskeleton driven by FSR strap consisting of 8 FSRs located at the forearm providing a passive and active mode of rehabilitation using a neural network for regression. In [21], there was a study on finding the optimal location for detecting hand gestures and wrist positions. The study included three possible positions, near the wrist, at the belly muscle at the forearm, and in the middle between these positions as shown in Fig. 10. It figured out that the middle position was the most suitable for capturing hand gestures and wrist position in terms of flexion and extension.

In the same year, Carlo et al. [87] used an FSR strap consisting of 8 FSRs located at the forearm, managed to evaluate a predictive model to predict human torques in three directions to provide a safer human–robot interaction system.

**Fig. 11:**
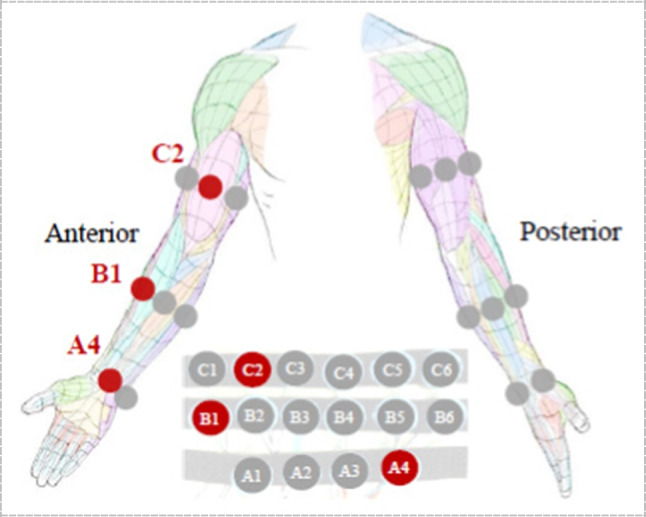
FSR armband positions

A recent study from Xiaohao Xu et al., discussed an algorithm for sensor placement optimization (SPO) [115]. To demonstrate this algorithm, they used three straps of a total of 16 force-sensitive resistors (FSR). One at the upper limb, the other at the bulk of the forearm and the last one at the wrist as shown in Fig. 11. Participants were monitored to perform eleven mechanical tasks as a simulation of the actual arm movements of a manufacturing worker. The signals are then extracted, processed, and classified using a semi-supervised technique called graph convolutional network (GCN).

Surprisingly, after using Graph-based Armband Modeling Network (GAM-Net) integrated with SPO algorithm, it figured out that only three sensing elements were sufficient to achieve accuracy of 90.8%, and for four sensing elements the accuracy was 93.1%.

## Signal processing

### FMG nature

**Fig. 12:**
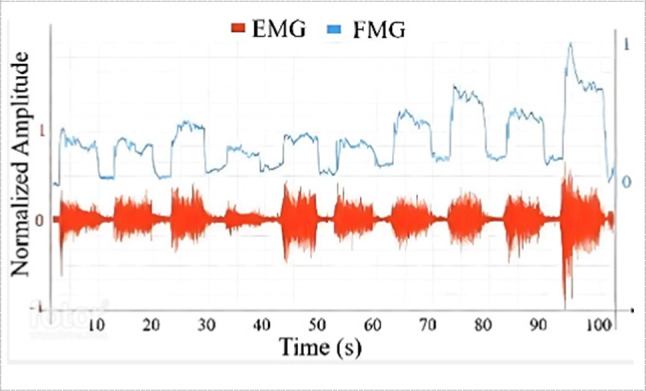
Comparison between FMG and EMG signals [51]

FMG signals extracted from different types of FSRs have different nature compared to EMG, which has a bipolar nature that centers about zero, while FMG reading is always positive [7, 11, 90, 111], Fig. 12. However, its nature gives it advantages in terms of processing and filtering. Since these signals correspond to volumetric changes in the targeted muscle, they are distinct enough to differentiate between wrist, hand, or finger gestures as claimed by [87].

**Fig. 13:**
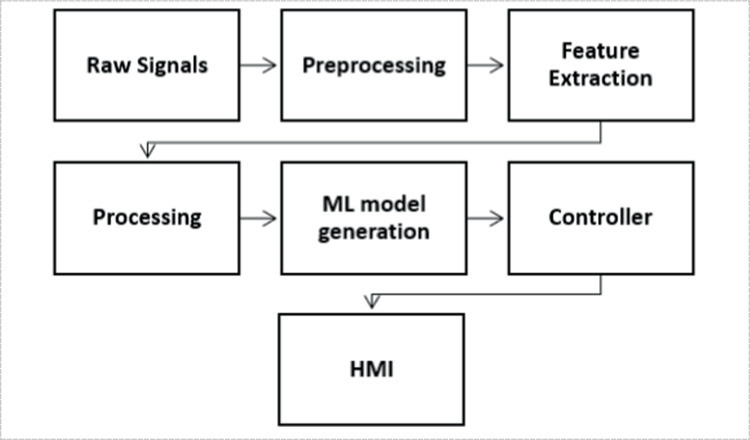
The full signal processing procedure

In the following sections, the whole process after acquisition FMG signals from FSR sensors till developing the machine learning model will be discussed Fig. 13.

### Preprocessing and feature extraction

After data were collected, there are some preprocessing steps to be taken, e.g., filtering or normalization, after which a model can be generated. At this section, preprocessing steps with feature extraction will be discussed.

Every single gesture has a distinctive FMG signal, [87]. These signals are represented either in a volt or digitized value. At this stage, directly after acquiring signals from sensors, raw FMG signals are available. Some literature used it directly for analysis and processing [22, 34, 35], achieving 91.2% and 83.5% in cross-validation and cross-trial evaluation schemes respectively as presented by Xianta Jiang et al. in [46].

**Fig. 14:**
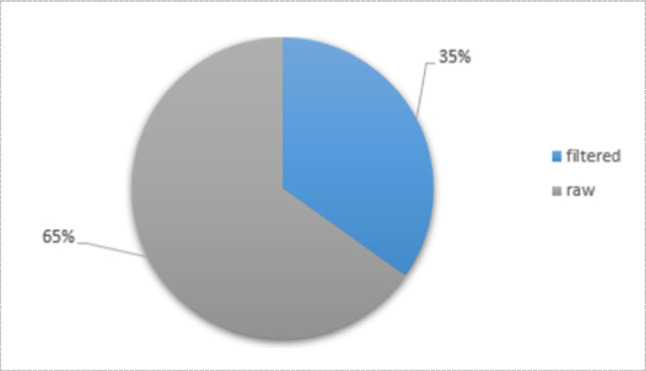
Percentage of literature used raw and filtered FMG signal ( number of publications = 66)

For further smoothing, some literature tends to use preprocessing steps such as filtering and normalization [24, 37, 58]. Michael et al. [106] used a low-pass filter with a 4 Hz cutoff frequency as well as Carlo et al. [85]. The cutoff frequency might be ranged from 4 to 20 Hz [111]. However, among numerous works in the literature (66 work of literature), a percentage of 65% used signals from FMG sensors in its raw form rather than using filtered signals Fig. 14.

It's of significance to highlight that some studies demonstrated comparable accuracy using raw signals without prior filtering. For instance, in [80], 99.7% accuracy was achieved in an eight-class experiment without applying signal filtering. In contrast, during in-session validation in [63], where preprocessing was employed, accuracy consistently exceeded 99%. This observation underscores the influence of signal filtering choices on classification performance.

Another preprocessing step where FMG signals are scaled from 0 to 1 based on the maximum and minimum value [7]. Otherwise, these signals could be auto scaled by subtracting the signal from its mean value and then divided by its standard deviation (SD). These numerical representations do not modify the signal pattern or affect the captured information.

#### Feature extraction

FMG feature extraction is a powerful method that not only ensure suitable signal analysis, but also minimize the dimensionality of the data space. It lowers the requirements for data storage and boosts the speed and efficiency of the machine learning algorithm [62].

Feature extraction is a procedure that is done over a number of signals to decode the motion of subjects with a suitable finesse level [6]. For EMG signals, there is a plenty of literature discussed the extracted features from either time or frequency or time–frequency domains [1, 32, 59, 73, 79]. While for FMG signals there are limited works of literature that discussed the extracted features for a specific application [3, 31, 42, 113].

In general, features are classified into two types: instance features and window-based features. In a series of multi-channel FMG signals, the instance feature is retrieved from a single instance, e.g., mean or standard deviation [6]. While the signal across a temporal frame is used to extract the window-based features, e.g., the mean magnitude of the signal and the average slope of a signal [111]. Commonly used features are summarized in Table 2.

**Table 2:** Extracted features

Feature	Abbreviation
Root mean square	RMS
Mean	–-
Standard deviation	SD
Mean of absolute deviation from the mean	MAD
Sum of absolute value	SAV
Mean of absolute value	MAV
Variance	VAR
Wavelength	WL
Window Symmetry	WS
Slope sign change	SSC
Simple square integral	SSI
Mean wavelet with db7	Db7
Difference absolute standard deviation value	DASDV
Average amplitude change	AAC
Log detector	LogD
Linear fit	CLF
Parabolic fit	CPF
Power spectral density	PSD
Likelihood	–-
Zero crossing	ZC
Square correlation coefficient	SCC
Coefficient of determination	${R}^{2}$
Skewness	–-
Normal mean square error	nRMSE
Mean square error	MSE

As presented by Xiao et al. [109], three features were extracted for eight input channels, root mean square, waveform length, and window symmetry besides raw FMG signals. In [113] seven statistical features were computed. Noting that the selected features to be extracted are dependent on the application requirements, and thus one cannot define a general feature set for FMG signals.

### Machine learning (ML)

A threshold value of FMG signal could be used to distinguish between two different states, e.g., extension and flexion of the wrist, for a constant elbow position. For different elbow configurations, it will be difficult to predict whether the wrist is flexing or extending. Here, it comes the value of machine learning in terms of providing a predictive model which can predict either torques values [87], hand gestures [7], finger movements [86] or gait monitoring [49], or controlling bio-robotic prosthetic devices [94] by training a set of data for generating a predictive model. It is considered as a powerful tool for extracting features from bio-signals [97].

In Machine learning, it is vital to guarantee that data are properly collected, and only important features are extracted. As a result, a more precise model will be generated. It can work properly under several conditions, providing the expected results and hence it can be integrated with an exoskeleton for real-life scenarios such as predicting a certain gesture or providing an assistive action in real-time.

There are two common machine learning techniques, classification, and regression. Both are used in many literature for a certain goal. Classification is used for providing a class for certain input, here the contribution of the ML model is just to identify the type of motion the patient is undergoing, such as recognize a certain static gesture. Meanwhile regression is used for predicting movements such as finger movements or wrist position. So, depending on the application, the type of the machine learning model is defined. However, both ensures an instantaneous action to provide an active rehabilitation for the patient.

Most common classification techniques used for FMG are linear discriminant analysis (LDA) [25, 48, 109, 110], support vector machine (SVM) [2, 23, 113] and artificial neural network (ANN) [77]. While most common regression techniques are support vector regression (SVR) [53, 88, 89], linear regression (LR) [58, 96], and general regression neural network (GRNN) [89]. Generally, the LDA technique has the advantage of ease of application in real-time processing and the ability to achieve comparable accuracies to other complex techniques, that is why it was used by many researchers in FMG applications [22, 123].

Using raw FMG signals but with the appropriate machine learning technique may contribute to further increasing the accuracy in predicting and effort the time used for some preprocessing steps. It has been proved by [25], that raw FMG signals processed with extreme learning machine (ELM) outperformed the raw FMG signals classified using artificial neural network (ANN) [65], support vector machine (SVM) [117], and linear discriminant analysis (LDA) [5, 26, 37]. The later methods can only achieve high accuracies with normalized FMG signals.

To generate a predictive model using a machine learning technique, data must be collected as much as possible. The more dataset, the more accurate will be the generated model. Some literature used 60% of the dataset for training and generating the model. The remaining 40% of the data were used for testing the generated model, others tend to use 80% for training and 20% for testing [7]. The ratio between data used for training and data used for testing is application dependent. If the target was real-time processing or real-time assessment, then less data used for training is suggested [117]. Meanwhile, if the target were to get an accurate model, more data are used for training than testing.

A recent study by [122], provides FMG datasets, their structures, and the collaborative tasks performed during the studies. It provides a good opportunity to give more attention to control design and develop a more robust ML model, by testing on these datasets, and avoid consuming time in collecting FMG data.

**Fig. 15:**
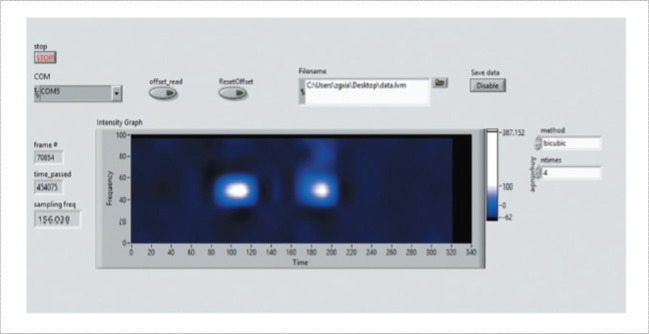
A custom LabVIEW interface for monitoring FMG signal pattern in real-time [114]

Most of the literature used MATLAB software (MathWorks) for processing and also for training and testing the predictive model [42, 60, 87, 93]. Data are transferred to MATLAB either by a Universal serial Bus (USB) [121] or Bluetooth module [42]. LabVIEW is usually used to provide a graphical user interface (GUI) to visualize real-time FMG signals tracking [17, 25] Fig. 15.

## Performance evaluation

Evaluating the performance of FMG machine learning model is a very important step, to assure that the wearable exoskeleton is performing as expected as for example. Since achieving high accuracies, ensures precise feedback and hence a precise control action. In the followings, difference between online and offline evaluation is discussed first, then most common performance coefficients are introduced.

### Online and offline evaluation

After the machine learning model is generated, it comes a necessary step to evaluate the model performance. Classification accuracy is one of the most common performance metrics to evaluate the performance of a given myoelectric pattern recognition algorithm [41, 76, 82]. It is a measure of how effective the machine learning technique is.

There are two methods to evaluate the model performance either offline or online (real-time). Some works of literature evaluated the accuracy offline by using pre-recorded data [110, 123], either for hand gesture recognition [25] or for estimating torque of wrist and forearm [87] or even in comparison between FMG and EMG signals in hand gesture classification [11]. Other works of literature evaluated the performance online for real-time pattern recognition [11, 40, 54] or for controlling robotic upper extremity prostheses [22, 105].

**Fig. 16:**
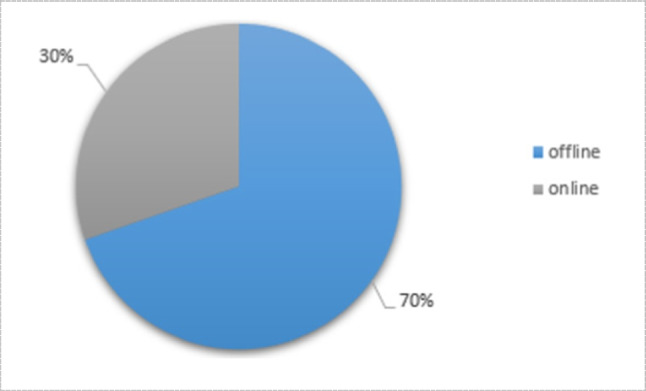
Percentage of literature used offline or online evaluation of accuracy (number of publications = 66)

Offline accuracy evaluation means dealing with pre-recorded data with no unexpected error during the evaluating process. Even if it achieves higher accuracies [4, 80, 87], it does not mean that it will be suitable for real-time applications [66]. Max et al. [76] evaluated offline myoelectric pattern recognition (MPR) performance metrics that can better relate to real-time controllability. It figured out that all the offline metrics failed to predict real-time decoding. Also, as discussed by [45], it has been found that high offline accuracy in classification is not always necessary to yield controllable systems, which clarifies the importance of online or real-time classification. It is worth noting that, online accuracy evaluation means that analysis is done when the data are captured (real-time analysis) which results in speedy result generation. In Fig. 16, it is shown percentage of the number of publications evaluated their accuracy either offline or online.

### Performance coefficients

There are many parameters and coefficients that literature adopted to evaluate their ML model performance. A common method used in evaluation is the N-fold cross-validation process. All data is randomly split into N folds. N-1 is used for training and the left fold is used for testing. e.g., tenfold cross-validation was used in [7] for a performance comparison between FMG and EMG in recognition of hand gestures, which is considered an accepted accuracy estimation method [57].

**Fig. 17:**
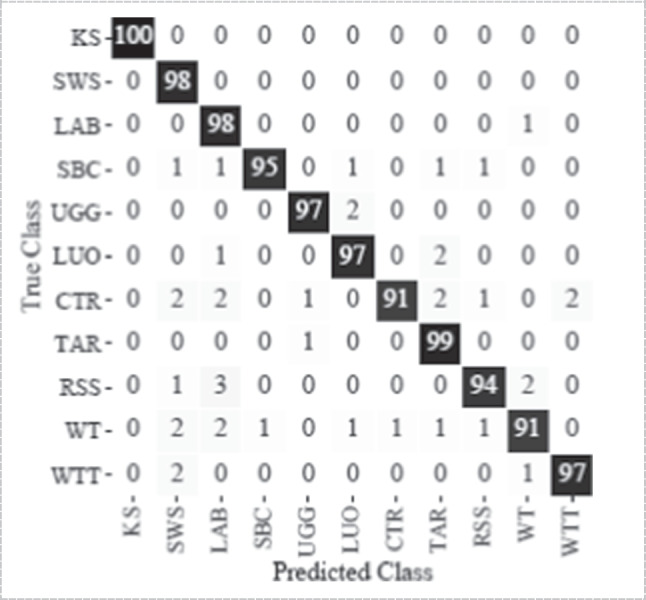
Confusion matrix

Confusion matrix is also used as an evaluation tool for machine learning techniques [33, 39, 100]. It is a table pattern that helps display the multiple outcomes of a classification problem's forecast and results. It displays the correct predictions (true positives and true negatives) and mistakes (false positives and false negatives) of a model Fig. 17. For instance, in a hand gesture recognition system using FMG, a confusion matrix helps assess its accuracy. True positives are correct hand gestures identified, true negatives are accurate rejections of unrelated motions, false positives are incorrect gesture detections, and false negatives represent missed hand gestures. This matrix aids in understanding the system's performance, ensuring it accurately recognizes hand movements for applications like sign language translation or gesture-controlled devices.

Another performance metric is the determination of coefficient ${R}^{2}$ which represents how well the model fits the data [43, 120]. The normalized root mean square error (NRMSE) also considered as an evaluation tool [16, 17, 74]. These two-accuracy metrics are used in [87, 88] for evaluating the performance of the regression or classification-based approach in order to compare between two models generated by two different machine learning techniques. Also, cross-trial accuracy was used in [47] for checking the accuracy of predicting seven ankle positions.

The motion test introduced by [77] was used for real-time evaluation. It requests the subjects to do different motions in a randomized manner while evaluating some key performance factors such as selection time, which is the time elapsed between the first forecast that differs from the others and the first right prediction. Another factor is completion time, which is the time consumed till the 20th right prediction. Completion rate, which is the proportion of movements that had 20 correct predictions before timeout. Finally, the real-time accuracy, which is expressed as a percentage of correct predictions over all forecasts made throughout the completion period.

Spearman’s Rho (R) is a non-parametric test to examine the relationship of two variables using a monotonic function [83], where |R|< 0.33, was considered a weak correlation, a medium range R value, 0.33 ≤|R|< 0.67, represented a moderate correlation, and 0.67 ≤|R| demonstrated a strong correlation.

## Discussion

There was a great effort from the research community in FMG approach, in designing customed FSR bands, studying a lot of factors from the sensor placements, and sensor types to machine learning techniques used in classifications or regressions. However, there are still uncovered points that represent challenges to provide more accurate models and precise signal acquisition which can adapt to real-life scenarios. Common points that directly affect the performance of an FMG system as overall or any robotic exoskeleton driven by FMG signals are discussed below.

One pivotal challenge arises from the inherent variability in sensor placement. In practical applications, it's an unrealistic expectation that FMG sensors will consistently occupy the exact same position each time. Furthermore, during prolonged usage, these sensors may naturally shift from their initial placement. Such variability can significantly impact signal accuracy, particularly when real-time processing is involved. In real-world scenarios, the reliability of FMG sensors is a critical concern.

FMG signals usually are not affected by electromagnetic interference, but noisy signals may appear in terms of unexpected pressures, such as being externally impacted. These unexpected pressures may produce misleading FMG signals, which is to be compensated during processing either by filtering or using intelligent processing techniques.

An additional layer of complexity arises from user-specific anthropometric measurements and their profound influence on signal quality. Parameters such as the ratio of wrist to forearm circumference, skinfold thickness to forearm circumference ratio, and grip strength play a substantial role in the accuracy of classifications and regressions. This issue has been meticulously explored in [25], shedding light on the significant impact of these anthropometric measurements. Notably, these measurements can be leveraged as valuable features for the construction of machine learning models, amplifying their importance in the FMG domain.

Generally, the variation of the FMG signal could be misleading if there was a problem in the signal acquisition process. Such a case means that the collected signals does not reflects the true physical procedure that the patient is undergoing. These variations, besides the abovementioned ones, may be also noticed at several levels. First, when muscles are stressed, their stiffness pattern changes, which results in a difference of the FMG signal. Second, everyone has a unique muscle form, that is when the muscle contracts, it has a distinctive shape of FMG signals. As a result, it may be difficult to generalize the generated machine learning model.

Deep learning techniques provide a highly effective and adaptive solution for the challenge of generalization within the context of rehabilitation and muscle activity analysis. These techniques hold immense promise due to their capacity to delve deeper into the nuanced aspects of muscle activity behavior. They can not only account for but also adapt to the diverse anthropometric measurements of individual users, thereby personalizing the rehabilitation process.

One of the notable strengths of deep learning is its ability to handle unforeseen and unanticipated inputs gracefully. This is particularly valuable in situations where unexpected errors or variations occur during the execution of a specific rehabilitation technique. Deep learning models, equipped with their inherent adaptability, can swiftly adjust, and continue to provide the most relevant and appropriate output, ensuring that rehabilitation procedures remain effective and safe.

Researchers can leverage these unique advantages to develop more versatile and universally applicable predictive models. By harnessing deep learning techniques, they can create predictive systems that are not only highly accurate but also robust in the face of real-world variations and unexpected scenarios. This empowers the field of rehabilitation to move towards a more comprehensive and user-centric approach, where everyone’s unique needs and responses are accommodated, ultimately leading to more effective and efficient rehabilitation strategies.

Moreover, it is crucial to acknowledge that the bulk of research in the FMG domain primarily involves individuals without preexisting muscle problems, as a result, there is a need for tests to be done on persons with limb impairments for further assurance of the validity of the generated predictive model.

Lastly, sensor fusion techniques have been gaining prominence as a potent approach to enhance the accuracy of predictive models. By integrating various sensing modalities, including Mechanomyography (MMG), surface electromyography (sEMG), and Force Myography (FMG), a more comprehensive understanding of muscle activity can be attained. For example, MMG excels in capturing high-frequency muscle vibration information, while FMG primarily focuses on low-frequency movement information. These approaches should be considered as complementary rather than mutually exclusive, representing distinct facets of muscle activity [41].

## Conclusion

This paper represents the main aspects of FMG technology starting from the nature of the signal and how to extract the signal, describing several types of sensors and their placement methodology passing through sampling rate to signal processing, where different features extraction and conditioning are discussed. Also, the difference between online and offline evaluation are highlighted and recent machine learning techniques used to summarize the whole processing procedure. Later, uncovered points are discussed highlighting the studies need to be conducted, presenting trendy terms in the field of rehabilitation using exoskeletons, such as deep learning and sensor fusion. We hope this survey paper paves the way for more enhancements in FMG approach.
